# Overview of genetic mutations causing adrenoleukodystrophy: A case-series study

**DOI:** 10.1016/j.ymgmr.2025.101237

**Published:** 2025-06-24

**Authors:** Mohadeseh Fathi, Sheyda Khalilian, Arezou Sayad, Parvaneh Karimzadeh, Farzad Ahmadabadi, Soudeh Ghafouri-Fard, Mohammad Miryounesi

**Affiliations:** a Student Research Committee, School of Medicine, Shahid Beheshti University of Medical Sciences, Tehran, Iran; b Department of Medical Genetics, Shahid Beheshti University of Medical Sciences, Tehran, Iran; c Genomic Research Center, Shahid Beheshti University of Medical Sciences, Tehran, Iran; d Department of Pediatric Neurology, Mofid Children Hospital, Shahid Beheshti University of Medical Sciences, Tehran, Iran

**Keywords:** Adrenoleukodystrophy, ABCD1, Iran

## Abstract

X-linked adrenoleukodystrophy (X-ALD) is a genetic disorder resulted from mutations in the *ABCD1* gene located at the Xq28 locus. This gene encodes a transporter protein responsible for importing very-long-chain fatty acids into peroxisomes. This research seeks to elucidate the clinical manifestations linked to various mutations in the *ABCD* gene among Iranian patients with X-ALD, considering the diverse severity of symptoms observed in this disease. Totally, six variants, including a novel variant (c.1781-47G > A) were identified in the *ABCD1* gene in the patients. All but one variant were classified as pathogenic or likely pathogenic; the remaining variant, c.1781-47G > A, was identified as a variant of uncertain significance. This study broadens the spectrum of *ABCD1* mutations among Iranian patients, providing applicable information for appropriate genetic counseling in the affected families.

## 1 Introduction

Peroxisomal disorders are a group of genetic conditions that arise from dysfunctions in peroxisomes, which are small organelles found in cells that play a crucial role in various metabolic processes. These disorders can be broadly categorized into two main types: single-enzyme deficiencies and peroxisomal biogenesis disorders [1]. The former category includes disorders that result from the deficiency of a specific enzyme within the peroxisome [2,3]. Each enzyme has a unique function, and when one enzyme is deficient, it can lead to the accumulation of toxic substances or the inability to metabolize certain compounds effectively [4]. Single-enzyme deficiencies include X-linked adrenoleukodystrophy (X-ALD). X-ALD results from mutations in the *ABCD1* gene located at the Xq28 locus, which encodes a transporter protein responsible for importing very-long-chain fatty acids (VLCFAs) into peroxisomes [5,6].

The resulting accumulation of VLCFAs can lead to adrenal insufficiency and progressive neurological decline [4]. The clinical spectrum of X-ALD ranges widely, from the rapidly progressive cerebral form (CALD) to milder adrenomyeloneuropathy (AMN), isolated adrenal insufficiency, or even asymptomatic cases [[7], [8], [9]]. CALD primarily manifests as cognitive decline accompanied by neurological impairments, including hemiplegia, quadriparesis, cerebellar ataxia, central auditory dysfunction, visual field deficits, and seizures. The condition progresses to a vegetative state within several years, ultimately leading to death. In contrast, AMN is a slow, noninflammatory axonopathy that predominantly damages the spinal cord's ascending sensory and descending motor tracts [9].

This research seeks to elucidate the clinical manifestations linked to various mutations in the *ABCD* gene, considering the diverse severity of symptoms observed in this disease.

## 2 Case presentation

This study was performed on five Iranian cases of X-ALD. Cases were referred to the Comprehensive Genomic Center, Tehran, Iran during 2016–2024 for molecular diagnosis and counseling.

Patients were eligible for genetic testing if they met one or more of the following clinical or biochemical features associated with ALD: Neurological manifestations (progressive cognitive/behavioral decline (suggestive of CALD), spinal cord dysfunction (typical of AMN), or peripheral neuropathy or cerebellar ataxia), Primary adrenal insufficiency, Family history of ALD, Elevated plasma VLCFAs, or suggestive MRI findings (Characteristic white matter abnormalities or spinal cord atrophy). Exclusion criteria were lack of biochemical (VLCFA) or clinical features suggestive of ALD, or confirmed alternative.

Informed consent forms were signed by patients or legal representatives of patients. All methods were performed in accordance with the Declaration of Helsinki. Related protocols were approved by ethical committee of Shahid Beheshti University of Medical Sciences.

## 3 Molecular diagnosis

First, DNA was retrieved from peripheral blood of the patients using the standard salting out method. WES was carried out using Illumina Novaseq6000 (Macrogen, Seoul, South Korea) with an average 100× coverage depth for >98 % of the targets. At that point, 100 paired-end base-pair reads were aligned to the human reference genome (GRCh37/hg19). In detail, raw FASTQ files were processed using BWA-MEM (v0.7.17) for alignment to the GRCh37/hg19 reference genome. Duplicate reads were marked with Picard (v2.23.8), and base quality score recalibration (BQSR) was performed using GATK (v4.1.8.1). Single-nucleotide variants (SNVs) and indels were called using GATK HaplotypeCaller, with joint calling across samples to improve sensitivity. Variants were filtered using GATK's recommended hard filters. Analytical sensitivity of the test was set at 97 % for SNVs and small insertions/deletions. Pathogenic and possible pathogenic variants were described based on the ClinVar database using the method commended by the Human Genome Variation Society (http://www.hgvs.org/). Variants classification was based on the guidelines of the American College of Medical Genetics. Clinical relevance (OMIM, ClinVar) and predicted pathogenicity (Combined Annotation Dependent Depletion [CADD] score > 20, rare population frequency [gnomAD MAF < 0.1 %]). Identified mutations were verified by Sanger sequencing (Codon genetics company, Iran). Given the homology between *ABCD1* (Xq28) and its pseudogenes (*ABCD1P1–P5*), we implemented stringent measures: WES reads aligning to *ABCD1* were visually inspected in IGV to confirm unique mapping (excluding reads with mismatches in pseudogene-homologous regions). All reported *ABCD1* variants were hemizygous in males (100 % allele frequency) or heterozygous in female carriers, as expected for X-linked variants. No low-level mosaicism was detected.

## 4 Results

This study included four males and a female. The males were hemizyous whereas the female (Case 5) had two variants. Except for one case, other cases were born to non-consanguine parents. Patients presented with diverse symptoms, particularly developmental regression. There was no report of adrenal insufficiency in any of cases. Totally, six variants, including a novel variant (c.1781-47G > A) were identified in the *ABCD1* gene in the patients. All but one variant were classified as pathogenic or likely pathogenic; the remaining variant, c.1781-47G > A, was identified as a variant of uncertain significance (VUS).

### 4.1 Detailed genetic and clinical findings in affected individuals

Case 1 was an 8-month-old male with fever, seizures, and developmental delays. Family history revealed a deceased sibling with leukodystrophy, supporting X-linked recessive (XLR) inheritance. Genetic testing revealed the hemizygous pathogenic variant *ABCD1* c.1849C > T (p.R617C) (NM_000033.4), classified as pathogenic (ClinVar-confirmed, rs4010613). This variant is well-characterized in ALD/AMN, necessitating urgent evaluation of male relatives for early intervention.

Case 2 was an 8-year-old male with ataxia, strabismus, speech regression, and white matter abnormalities on MRI. Genetic testing demonstrated the hemizygous likely pathogenic variant *ABCD1* c.1538 A > G (p.K513R) (NM_000033.4), not yet reported in ClinVar.

Case 3 was an 8.5-year-old male with developmental regression and clinical ALD diagnosis. This patient was hemizygote for likely pathogenic splice-site variant *ABCD1* c.1781-1G > C (NM_000033.4). *In silico* tools predicted disruption of the canonical splice site, supporting pathogenicity.

Case 4 was a 6.5-year-old male from a consanguineous family, presenting with speech regression and limb weakness. He had hemizygous likely pathogenic variant *ABCD1* c.839G > C (p.R280P) (NM_000033.4) with conflicting ClinVar interpretations. Variant interpretation complicates counseling in this case.

**Fig. 1:**
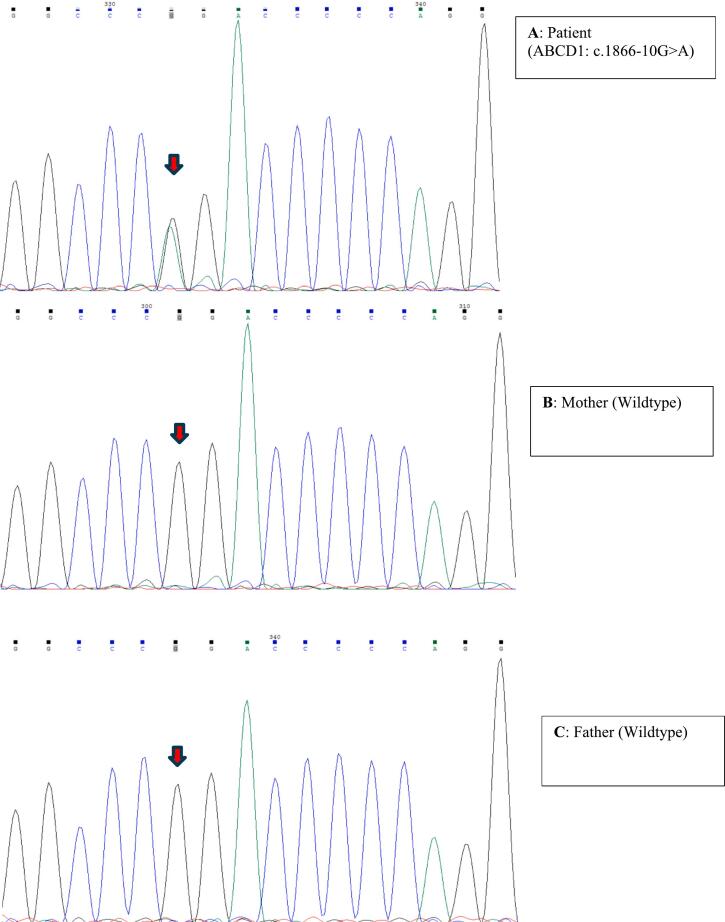
Chromatogram of Sanger sequencing results for confirmation of *de novo* origin of the c.1866-10G > A variant in Case 5.

Finally, Case 5 was a 7-year-old female with developmental regression, hearing loss, and leukodystrophy. Genetic findings were as follow: Pathogenic/Likely Pathogenic: *ABCD1* c.1866-10G > A (heterozygous, rs398123108) and *ABCD1* c.1781-47G > A (VUS, rs782363851). Notably, the c.1866-10G > A variant occurred *de novo* (Fig. 1).

Inherited from her mother, the c.1781-47G > A variant was present in her maternal uncle, who remained symptom-free despite the genetic finding. An additional finding in this case was *HUWE1* c.6030 + 1G > A (NM_031407.7), a likely pathogenic variant linked to X-linked intellectual disability (Turner type). The clinical manifestations in this case were more compatible with CALD.

Table 1 shows the summary of clinical and molecular findings in the patients.

**Table 1:** Summary of clinical and molecular findings in Iranian patients with adrenoleukodystrophy (ALD).

Case number	Gene	Transcript⁎	Variant	Associated disease	OMIM	Inheritance	Zygosity	ACMG classification	Clinvar	dbSNP rsID	Age	Sex	Consanguinity	Indication
1	*ABCD1*	NM_000033.4	c.1849C > T p.R617C	ALD ALD, adult	300100 300100	XLR XLR	Hemi	Pathogenic	Pathogenic	rs4010613	8 months	Male	No	Fever, seizure, developmental delay. First child died due to leukodystrophy.
2	*ABCD1*	NM_000033.4	c.1538 A > G p.K513R	ALD ALD, adult	300100 300100	XLR XLR	Hemi	Likely Pathogenic	NR	–	8 years	Male	No	Ataxia, strabismus, speech regression and white matter abnormality in brain MRI
3	*ABCD1*	NM_000033.4	c.1781-1G > C	ALD ALD, adult	300100 300100	XLR XLR	Hemi	Pathogenic	Pathogenic	–	8.5 years	Male	–	Developmental regression and clinical diagnosis of leukodystrophy
4	*ABCD1*	NM_000033	c.839G > C p.R280P	ALD ALD, adult	300100 300100	XLR XLR	Hemi	Likely Pathogenic	Conflicting	–	6.5 years	Male	Yes	Speech regression, weakness of hand and foot with clinical diagnosis of leukodystrophy
5	*ABCD1*	NM_000033.4	c.1866-10G > A	ALD ALD, adult	300100 300100	XLR XLR	Het	Pathogenic	Pathogenic/Likely pathogenic	rs398123108	7 years	Female	No	Developmental regression, hearing impairment, and leukodystrophy
			c.1781-47G > A				Het	VUS	NR	rs782363851				
	*HUWE1*	NM_031407.7	c.6030 + 1G > A	Intellectual developmental disorder, X-linked syndromic, Turner type	309590	XL	Het	Likely Pathogenic	NR	–				

⁎ Human reference genome: GRCh37/hg19.

## 5 Discussion

In an attempt to find the genetic background of X-ALD, we used WES technique in five Iranian patients presented with different manifestations suggestive of this disorder. All patients had cerebral ALD which was characterized by progressive behavioral, cognitive, and neurologic defects. Onset of symptoms ranged from infancy to childhood. Totally, we found six variants. Checking with the *ABCD1* Variant Registry (https://adrenoleukodystrophy.info/mutations-and-variants-in-abcd1), we found that c.1781-47G > A is a novel variant. The c.1849C > T variant has been repeatedly identified in ALD patients in different studies [7,[10], [11], [12]]. Notably, this variant has been associated with no detectable ALD protein (ALDP) in patient cells [7]. Similarly, the c.1538 A > G has been previously identified in 3 ALD cases [9,13] with no detectable ALDP in patient cells (https://adrenoleukodystrophy.info/mutations-and-variants-in-abcd1). While not being specifically mentioned, the main clinical and laboratory manifestation of the patients with different variants, including the c.1849C > T and c.1538 A > G variants have been vision and hearing loss, unsteady gait, skin pigmentation, intellectual regression, elevated VLCFA levels and adrenal insufficiency [13].

The c.1781-1G > C variant has been reported in a single ALD case and in ALD newborn screening [14], affecting ALDP function in fibroblasts [14]. The diagnosis of ALD has been confirmed in this newborn by elevated levels of VLCFAs, including C26:0-lysophosphatidylcholine (C26:0-LPC) [14]. The c.839G > C variant has been previously reported in an Iranian patient with X-ALD suffered from vomiting and impaired adrenocortical function [15].

We encountered a diagnostic challenge in Case 5 who carried a pathogenic variant beside a VUS in *ABCD1*, the latter being detected in her unaffected uncle. If only one of the variants is pathogenic (presumably c.1866-10G > A) or the variants are *in cis*, the disease manifestation might be due to skewed X-inactivation. In line with this hypothesis, a single report has presented a female case of CALD with asymmetric demyelination of bilateral white matter in brain MRI, elevated levels of plasma VLCFAs, and an *ABCD1* c.919C > T (p.Q307X) heterozygous pathogenic mutation, which was inherited from the asymptomatic mother. Further analyses have revealed almost complete inactivation of the normal paternal X chromosome in this case [16]. Alternatively, co-occurrence of *ABCD1* and *HUWE1* variants might lead to this pathology, requiring tailored management.

In brief, the mentioned variants in the *ABCD1* gene were associated with the CALD in the Iranian patients. It is worth mentioning neither plasma VLCFA levels nor the type of pathogenic *ABCD1* variants reliably predict disease phenotype or progression in ALD. While these markers confirm diagnosis, they do not correlate with clinical severity, age of onset, or organ involvement (*e.g.*, cerebral demyelination *vs.* adrenomyeloneuropathy) [[17], [18], [19]]. In fact, the same pathogenic variant can be associated with each of the known subtypes [7,10]. Even the same phenotype could be detected both with large deletions and with missense pathogenic variants [17].

While the variants in the *ABCD1* gene are responsible for the main manifestation of X-ALD, there is no association between the nature of *ABCD1* variants and the clinical manifestations, and the molecular mechanism of phenotypic variability in this disorder has not been clarified. In fact, modifier genes can influence the severity or progression of X-ALD, including adrenal insufficiency [20]. For instance, APOE4 might be associated with worsening of cerebral disease in ALD, serving as a modifier of phenotype severity [21].

The presence of pseudogenes in the human chromosome regions 2p11, 10p11, 16p11, and 22q11 with high similarity with *ABCD1* gene [22] complicates molecular diagnosis of X-ALD. Attempts have been made to design PCR primers for PCR-sequencing that do not amplify the pseudogenes [13]. Moreover, particular attentions should be made for interpretation of the WES results.

The spectrum of *ABCD1* mutations among Iranian patients has been assessed in various studies. For instance, an *ABCD1* mutation (c.253dup leading to p.Arg85Profs*110) was detected in 35 patients (out of 96 pedigree members) among an extended pedigree among Lurs, suggesting high prevalence of X-ALD among consanguineous Lurs [23]. This variant might be a founder mutation among Lurs. Another study in four pedigrees led to identification of a previously known missense mutation (c.1978C > T) and three novel mutations (c.1797dupT, c.879delC, c.1218C > G) in the *ABCD1* gene among Iranian patients [24]. In addition, c.253_254insC (p.R85Pfs112*) was reported in five affected patients in an expanded pedigree of Iranian patients [25].

Comprehensive analysis of 406 X-ALD mutations has shown a variety of different mutations, including missense (*n* = 227), frameshift (*n* = 110), nonsense (*n* = 37), small in-frame insertions or deletions (*n* = 16), and large deletions (n = 16). Notably, most of X-ALD families had a unique mutation and a total of 234 mutations have been non-recurrent mutations [7]. A more recent update on *ABCD1* variants has confirmed the previous report about the order of pathogenic variants frequency, and global clustering of disease-causing variants in exons 1–2 and 6–9 [17].

The spectrum of *ABCD1* mutations identified in this cohort of Iranian patients and other reports from this population suggest the genetic heterogeneity of X-ALD in this population. While comprehensive prevalence data for X-ALD in Iran are currently limited, our findings underscore the significance of family-based genetic screening for early diagnosis in at-risk relatives, particularly given the therapeutic implications for presymptomatic males. Hematopoietic stem cell transplantation, when performed early in the disease course, can improve patients' prognosis in cerebral ALD. Therefore, in families with a confirmed *ABCD1* mutation, cascade genetic testing of male relatives—combined with biochemical monitoring (such as VLCFA analysis) and neuroimaging—may permit timely intervention. Future population-based studies are needed to establish the prevalence of X-ALD in Iran and to optimize cost-effective screening strategies (such as targeted testing for recurrent mutations). Until such data are available, clinical alertness and familial screening remain practical approaches to mitigate disease burden.

## CRediT authorship contribution statement

**Mohadeseh Fathi:** Investigation, Data curation, Conceptualization. **Sheyda Khalilian:** Investigation, Data curation, Conceptualization. **Arezou Sayad:** Investigation. **Parvaneh Karimzadeh:** Investigation. **Farzad Ahmadabadi:** Investigation. **Soudeh Ghafouri-Fard:** Writing – review & editing, Writing – original draft, Supervision. **Mohammad Miryounesi:** Supervision, Conceptualization.

## Declaration of competing interest

None.

## Data Availability

The datasets generated and/or analysed during the current study are available in the Clinvar repository (https://www.ncbi.nlm.nih.gov/clinvar/?term=%22abcd1%22%5BGENE%5D).
